# A Novel VHH Antibody Targeting the B Cell-Activating Factor for B-Cell Lymphoma

**DOI:** 10.3390/ijms15069481

**Published:** 2014-05-28

**Authors:** Wen Wu, Shenghua Li, Weijing Zhang, Jian Sun, Guangda Ren, Quanchao Dong

**Affiliations:** 1 Department of Lymphoma, Affiliated Hospital of Academy of Military Medical Sciences, Beijing 100071, China; E-Mail: wuwen1981@163.com; 2 Department of Blood Transfusion, Jinan Military General Hospital, Jinan 250031, China; 3 Tianjin International Joint Academy of Biotechnology and Medicine, Tianjin Shengfa NabioTech Co., Ltd., Tianjin 300457, China; E-Mail: haoyou3003@gmail.com; 4 Department of Molecular and Cellular Pharmacology, School of Pharmaceutical Science and Technology, Tianjin University, Tianjin 300072, China; 5 Division of Science Popularization, China Science and Technology Exchange Center, Beijing 100045, China; E-Mail: dongqc@cstec.org.cn

**Keywords:** immune VHH phage display library, B cell-activating factor, sdAb

## Abstract

Objective: To construct an immune alpaca phage display library, in order to obtain a single domain anti-BAFF (B cell-activating factor) antibody. Methods: Using phage display technology, we constructed an immune alpaca phage display library, selected anti-BAFF single domain antibodies (sdAbs), cloned three anti-BAFF single-domain antibody genes into expression vector pSJF2, and expressed them efficiently in *Escherichia coli*. The affinity of different anti-BAFF sdAbs were measured by Bio layer interferometry. The *in vitro* biological function of three sdAbs was investigated by cell counting kit-8 (CCK-8) assay and a competitive enzyme-linked immunosorbent assay (ELISA). Results: We obtained three anti-BAFF single domain antibodies (anti-BAFF64, anti-BAFF52 and anti-BAFFG3), which were produced in high yield in *Escherichia coli* and inhibited tumor cell proliferation *in vitro*. Conclusion: The selected anti-BAFF antibodies could be candidates for B-cell lymphoma therapies.

## 1. Introduction

B cell-activating factor (BAFF) also known as B lymphocyte stimulator (BLyS) is a member of the tumor necrosis factor (TNF) ligand super-family of cytokines [1], and is expressed and secreted by monocytes, DCs, neutrophils, basophils, stromal cells, activated T cells, epithelial cells, and malignant B cells [2]. BAFF binds B cells via three receptors: BAFF-R, TACI (transmembrane activator and calcium modulator ligand interactor) and BCMA (B cell maturation antigen). Binding stimulates B cell proliferation and promotes their survival [3,4] through activation of the alternative NF-κB pathway [5,6]. Over-expression of BAFF results in peripheral B cell numbers increasing significantly and may be involved in autoimmune disease and B cell malignancies [7].

Clinical studies have demonstrated that BAFF plays crucial roles in B-cell lymphoma [8]. First, BAFF levels both in Hodgkin’s lymphoma (HL) and non-Hodgkin’s lymphoma (NHL) patients are significantly elevated compared with those of healthy controls [9,10]. Serum BAFF levels of follicular non-Hodgkin’s lymphatic tumors (follicular lymphoma, FL) are 3–4 times higher than normal levels. Furthermore, BAFF and its receptors are highly expressed in reactive lymphoid tissues and B-cell lymphomas, including diffuse large B-cell lymphoma (DLBCL), primary central nervous system lymphoma (PCNSL), mantle cell lymphoma (MCL), marginal zone B-cell lymphoma (MZBCL) and plasma cell myeloma (PCM). Second, the expression of BAFF is correlated with Ann Arbor stage [11]. BAFF mRNA levels in early B-NHL patients are significantly lower than those in patients with advanced B-NHL. Lastly, BAFF levels correlate with disease severity, response to therapy, and prediction of clinical outcome. Serum BAFF levels are correlated with responses to initial therapy, progression-free survival (PFS), and overall survival (OS). A high level of BAFF is associated with several poor prognostic characteristics. Patients with a high serum BAFF level at the time of diagnosis had a significantly worse median OS compared to those with a low serum BAFF level, and patients with high serum BAFF also had a lower CR rate than those with low BAFF levels and a shorter median PFS.

Currently, some strategies are designed to eliminate B cells through the use of anti-BAFF antibodies [12] when elevated levels of BAFF are found in patients with autoimmune disease or B cell malignancies. However, the majority of approved antibodies use full-length IgG, which comes with certain limitations, including the immunogenicity of murine molecules [13], inability to reach the cryptic site desired for efficacy [14], distribution to normal organs, poor penetration of solid tumors, and difficulty of expression and purification. Along with progress in genetic engineering techniques, there have been major efforts to obtain smaller antibodies to cope with these shortcomings. Here, we describe a single variable domain of heavy-chain antibody only IgG from camels (HCAbs) [15].

Heavy chain antibodies from camelid species lacking light chains and CH1 domain formed the basis for generating functional single-domain antibody fragments (sdAb) from their variable domains (VHH). The molecular weight of VHH is 15 kDa, much smaller than other recombinant antibody formats (60 kDa), hence it has also been called a nanobody. The smaller size results in lower immunologic response and better pharmacokinetics. VHH not only has a smaller size but also superior properties. There are only three complementarity-determining regions (CDRs) of VHH [16,17], and they are somewhat unique: the CDR1 and CDR2 loops are canonical in structure, and an unusually long surface CDR3 fold enables VHH to recognize alternative antigen epitopes and enhances solubility [18,19]. VHH is highly stable in extreme pH conditions, can bind to their target at high concentrations of chaotropic agents and is easily produced in large quantities [20]. Because of those favorable properties, VHHs have been given much attention by researchers and have been used in settings ranging from scientific research to clinical detection and treatment. In an early study, researchers derived VHH agents from non-immune libraries, but immune libraries were subsequently found to lead more directly to VHHs with higher affinities [21].

The aim of the current study was to obtain single-domain anti-BAFF camel antibodies from an immune library to improve clinical efficacy of B cell malignancies. Here, we detail the construction of an immune library as well as panning, expression and characterization of functional sdAbs with high specificities and sensitivities to BAFF.

## 2. Results

### 2.1. Construction of the Immunized Alpaca Library

A young alpaca was immunized subcutaneously four times with BAFF, and the antigen-specific immune response was tracked by enzyme-linked immunosorbent assay (ELISA). The total anti-BAFF IgG serum titer reached 1:10,000 at 3 months after the first immunization. Alpaca lymphocytes were collected from EDTA-treated blood, total RNA was extracted from 10^6^ lymphocytes, and cDNA was prepared and used as the template to amplify *VHH* genes. A 500 bp product was observed in agarose gels, indicating successful amplification of the antibody heavy chain, and the gel purified *VHH* genes were served as the template for amplification of the variable domains. The polymerase chain reaction (PCR) fragments were integrated into phage vector pHEN-6 using SfiI sites introduced in the primers. The recombinant plasmids were validated by colony PCR and then transformed in TG1 cells. A VHH library of approximately 3 × 10^9^ independent clones was constructed. To determine the percent of clones carrying *VHH* genes, PCR was performed on 60 clones selected randomly from the library. Colony PCR showed that 90% of this VHH library contained a phagemid insert of a size corresponding to a VHH.

### 2.2. Selection of Alpaca Heavy Chain Variable Domains (VHHs) against B Cell-Activating Factor (BAFF)

We panned the immunized alpaca phage display library for anti-BAFF sdAbs over the course of three rounds of selection and amplification of the bound phage. The titer of the eluted phage increased significantly after the last round of panning, indicating that the library was already enriched in BAFF-specific binders (Table 1). More than half of the clones bound strongly to BAFF but not to a control bovine serum albumin (BSA) in the phage ELISA.

**Table 1:** Enrichment of specific phages during subsequent rounds of panning.

Round	Titer of input phage	Titer of output phage	Output phages/input phages
1	1.3 × 10^12^	1.48 × 10^6^	1.14 × 10^−6^
2	7.3 × 10^11^	4.03 × 10^7^	5.52 × 10^−5^
3	6.3 × 10^11^	1.70 × 10^7^	2.70 × 10^−5^

### 2.3. Sequence Alignment of VHHs

**Figure 1:**
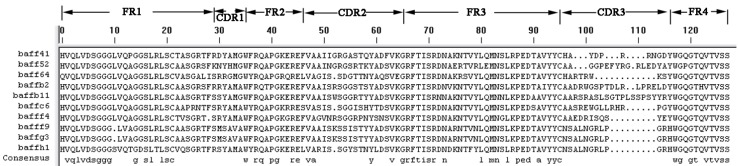
Comparative alignment of the amino acid sequences of anti-BAFF (B cell-activating factor) sdAbs, showing four hallmark amino acid changes at positions F(Y) 37, E44, R45 and G47. The cysteine positions in complementarity-determining region 1 (CDR1) and CDR2 are fixed at between 31–35 and 45–67, respectively. The position of cysteine in CDR3 varies.

Ten clones were sequenced and showed significant diversity (Figure 1). Of these 10 clones, BAFF52, BAFF64 and BAFFG3 yielded the highest ELISA signals and were therefore selected for further characterization.

### 2.4. Expression of Soluble VHH

**Figure 2:**
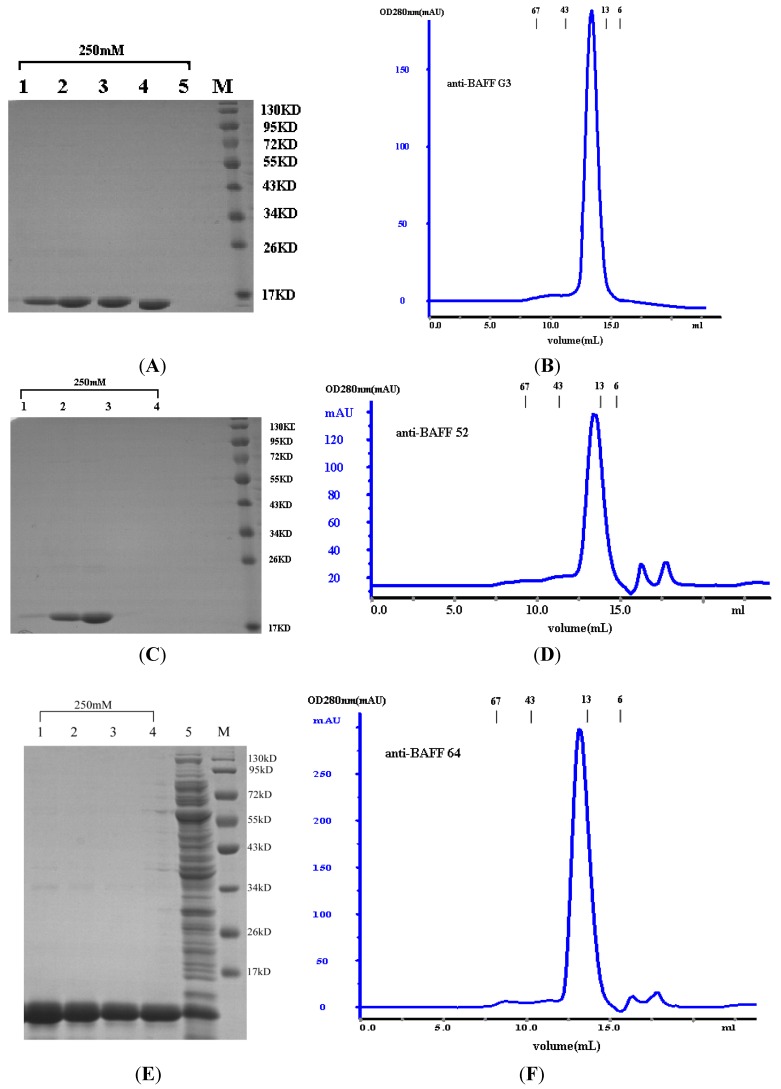
Sodium dodecyl sulfate-polyacrylamide gel electrophoresis (SDS-PAGE) and size exclusion chromatography of VHHs. BAFF, B cell-activating factor. (**A**) SDS-PAGE analysis of expression and purification anti-BAFFG3. Lane M: Molecular weight protein marker; Lane 1–5: The purified products collected from different test tubes in the same batch; (**B**) Purified anti-BAFFG3 protein was analyzed by size exclusion chromatography with a Superdex^75^ column; (**C**) SDS-PAGE analysis of expression and purification anti-BAFF52. Lane M: Molecular weight protein marker; Lane 1–4: The purified products collected from different test tubes in the same batch; (**D**) Purified anti-BAFF52 protein was analyzed by size exclusion chromatography with a Superdex^75^ column; (**E**) SDS-PAGE analysis of expression and purification anti-BAFF64. Lane M: Molecular weight protein marker; Lane 1–4: The purified products collected from different test tubes in the same batch; Lane 5: Total cells of TG1 with pSJF2-*VHH* induced by 1 mM isopropyl β-d-thiogalactopyranoside (IPTG) for 4 h before purified; (**F**) Purified anti-BAFF64 protein was analyzed by size exclusion chromatography with a Superdex^75^ column.

*VHH* genes were amplified and cloned into the pSJF2 expression vector. BAFF52, BAFF64 and BAFFG3 clones were solubly expressed in the periplasmic space of TG1. The purity of proteins was assessed by sodium dodecyl sulfate-polyacrylamide gel electrophoresis (SDS-PAGE; 12% acrylamide) after purification. As seen in Figure 2A,C,E, three sdAbs migrated at an expected size of 15 kDa under reducing conditions. To confirm protein purity, the three sdAbs were passed through a Superdex^75^ gelfiltration column (GE Healthcare Bio-Sciences AB, Uppsala, Sweden). No degraded or aggregated products were observed using either technique as the 3 sdAbs eluted from the Superdex^75^ column as a single peak and migrated as single bands under reducing and non-reducing SDS-PAGE. On a calibrated Superdex^75^ column anti-BAFF64 anti-BAFF52, anti-BAFFG3 eluted from 12.69 to 13 mL, equivalent to 15 kDa (Figure 2B,D,F). The yield of purified anti-BAFF64 was approximately 25 mg/L, and anti-BAFFG3 and anti-BAFF52 yielded approximately 20 mg/L.

### 2.5. Antibody Affinity Constant Measurement

The affinities of the purified VHHs were then analyzed by BioLayer interferometry (BLI) using Forte Bio’s Octet System (Pall Forte Bio Europe, Portsmouth, UK). Anti-CD20 sdAb was the negative control. The resultant fitted kinetic data are shown in Table 2. The kinetic analysis revealed that the anti-BAFF52 has a computed *K*_d_ of 3.18 × 10^−6^ M, whereas anti-BAFF64 and anti-BAFFG3 have higher affinities, reflecting higher association rates and lower dissociation rates. The lower affinity of anti-BAFF52 may be improved by affinity maturation [22] or by constructing a divalent form of the diabody with a similar binding affinity to the whole antibody.

**Table 2:** The relative affinity of selected antagonistic anti-BAFF (B cell-activating factor) sdAbs as measured by BioLayer interferometry (BLI) using Forte Bio’s Octet System.

SdAbs	KD (M)	kon (1/Ms)	kdis (1/s)
Anti-BAFF64	3.19 × 10^−7^	2.29 × 10^3^	7.29 × 10^−4^
Anti-BAFF52	3.18 × 10^−6^	5.71 × 10^3^	1.82 × 10^−2^
Anti-BAFFG3	8.23 × 10^−7^	2.90 × 10^3^	2.39 × 10^−3^

All constants were calculated from five data sets obtained with different concentrations of VHHs (from 1.25 to 20 µM) by a global Langmuir 1.1 method.

### 2.6. Antigen Binding Specificity of the Purified VHH

**Figure 3:**
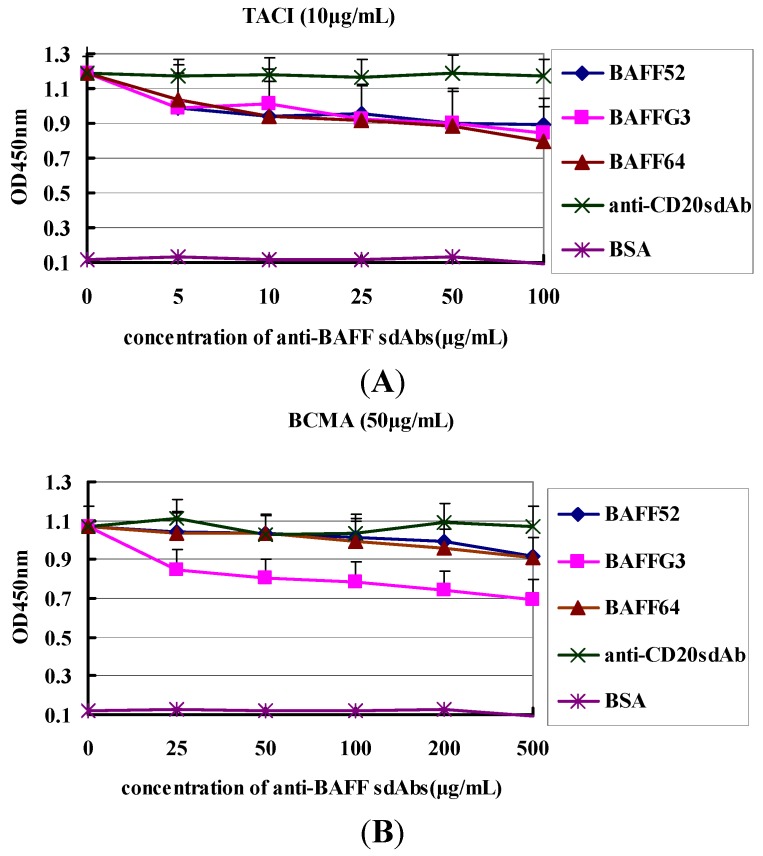
Competitive binding of VHHs for TACI (transmembrane activator and calcium modulator ligand interactor) and BCMA (B cell maturation antigen). Competitive enzyme-linked immunosorbent assay (ELISA) was used to assess competition across a range of concentrations. (**A**) Inhibition of TACI by sdAbs; (**B**) Inhibition of BCMA by sdAbs. Anti-CD20 sdAb was the negative control.

Effective competitive inhibition depends on the relative affinity and concentration of both the antibody and ligand. TACI and BCMA are two natural BAFF receptors, which may be used to detect the specificity of the VHHs and map the epitope. The three sdAbs were therefore tested for competition with BCMA and TACI. Compared with 6% of the negative control (anti-CD20 sdAb), the inhibition ratios of the three sdAbs significantly increased with sdAbs concentrations. The inhibition ratio of anti-BAFFG3 (500 μg/mL) to BCMA was 33%, and the inhibition ratio of anti-BAFF64 (100 μg/mL) to TACI was 31% (Figure 3). The results indicated that the three VHHs eluted by BCMA and TACI competition may have different epitopes on BAFF.

### 2.7. Efficiency of Inhibition of Tumor Cell Proliferation in Vitro

**Figure 4:**
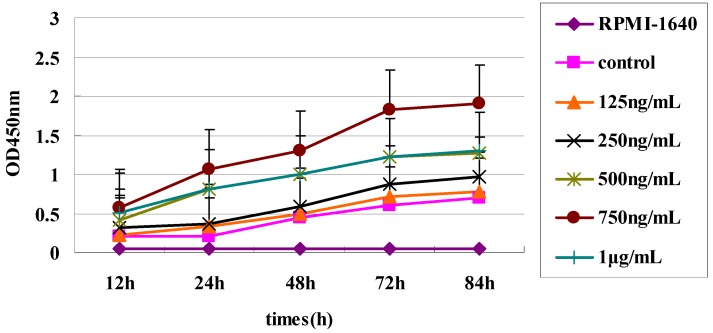
Cell growth curves of the Raji cell line. Raji cells were grown in 96-well plates at 37 °C in Roswell Park Memorial Institute (RPMI)-1640 (GibcoBRL, Gaithersburg, MD, USA) with calf serum and five different concentrations of BAFF (125 ng/mL to 1 μg/mL). Measurements were taken at 12, 24, 48, 72 and 84 h. The negative control used only RPMI-1640 without BAFF. The number of Raji cells detected by CCK-8 assay was highest when incubated with BAFF (750 ng/mL) for 72 h.

**Figure 5:**
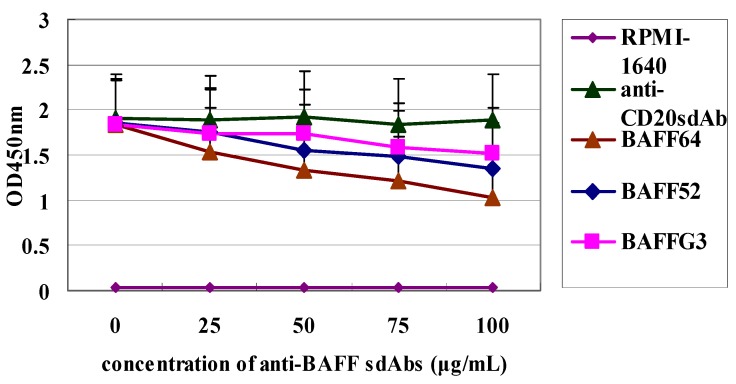
Inhibition of Raji tumor cell proliferation by three sdAbs. Cell viability was assayed by the CCK-8 method. Anti-CD20 sdAb was the negative control. The effects of VHHs on treated Raji cells were presented as the inhibitory ratio compared to the untreated group. The inhibition ratio of BAFF64 (100 μg/mL) was 55%.

The inhibitory effect of the three sdAbs on the proliferation of the Raji cell line was investigated by CCK-8 assay. Raji cells were incubated with BAFF (750 ng/mL) for 48 h (Figure 4), and then three sdAbs were added at a range of concentrations. The results showed that all three sdAbs could significantly inhibit Raji cell proliferation (Figure 5). The suppressive effect of anti-BAFF64 was much higher than other sdAbs, and it may be binding at a site critical to BAFF function.

## 3. Discussion

The purpose of this study was to generate anti-BAFF sdAbs and to determine their biological effects. To the best of our knowledge, this is the first report of the effects of anti-BAFF sdAbs with B-cell lymphoma cells. In the present study, we have successfully constructed the immunized alpaca sdAb library and isolated three anti-BAFF sdAbs (anti-BAFF64, anti-BAFF52 and anti-BAFFG3) from the phage library. Tests of their biological effects showed that the three sdAbs were high affinity and inhibited tumor cell proliferation *in vitro*, providing a promising opportunity for the development of new B-cell lymphoma therapeutics.

First, we constructed the immunized alpaca VHH library. The choice of phage display strategy depends on the required affinity and the target application of the desired antibody. In order to isolate sdAbs with high affinities for BAFF, with possible therapeutic applications in B-cell lymphoma, we chose to construct an immunized alpaca VHH library to produce anti-BAFF sdAbs. An immune phage antibody library has many advantages. First, the immune phage antibody library contained a large number of antigen-specific antibodies, some of which will also have been affinity matured by the immune system [23,24]. High-affinity antigen binding domains can be isolated by screening relatively few clones from immune libraries; Second, the immune response removes irrelevant antibodies, thus improving library quality before cloning starts. Animals can be made tolerant for certain irrelevant antigens, after which a mixture of the relevant and irrelevant antigens is used for immunization [25]; Third, VHH libraries from immunized alpaca retain full functional diversity. The young alpaca was immunized with BAFF four times over more than two months, and the titer was tested immediately after the serum was obtained. The antibody titer of the immune serum against BAFF was a minimum of 1.992 after the fourth injection, as opposed to the normal serum’s titer of 0.1. Alpaca possesses two types of antibodies, conventional antibodies and a heavy chain IgGs (HCAbs), which account for 50% of alpaca sera. The heavy chain IgGs include two types, IgG2 and IgG3. IgG2, a homodimer of 46 kDa chains with an extended hinge could substitute for the CH1 domain, only binds to protein A. In contrast, IgG3, which consists of two chains of 43 kDa, binds to protein A and G after reduction. The antigen-specific heavy chain antibodies can therefore be determined by the presence of proteins A and G. *VHH* genes were cloned from alpaca lymphocytes by nested PCR. VHH cloning was repeated several times to increase the number of *VHH* genes in the library. Obviously, a larger the number of different VHH clones that bind BAFF antigen will increase the likelihood of finding a VHH with the specific properties that are most important to us. More than 11 pairs of different primers were selected to amplify *VHH* genes in the first round of PCR, so that the different degenerate primers might amplify different antibody heavy chain genes, further increasing the diversity of the VHH library. Amplified *VHH* genes were purified, digested, and ligated into phage vector pHEN-6. The recombinant plasmids were introduced and electroporated into *Escherichia coli* TG1. Phage was precipitated with polyethylene glycol (PEG)/NaCl (20% PEG6000–2.5 M NaCl), and the phage titer was calculated. The resultant immunized alpaca VHH library contained 3 × 10^9^ members.

Secondly, the single-chain antibody recognizing BAFF was selected from the immune phage library by panning. Equally important to immune phage library construction is the design of an appropriate selection strategy. Effective panning and gradual decreasing of the concentration of BAFF antigen resulted in the isolation of the most specific VHHs during each panning. Streptavidin-coated microbeads were used to capture the biotinylated antigen. Due to the high binding affinity of streptavidin to biotin, the interaction between the phage particle and antigen takes place in the solution, which permits precise control of the antigen concentration and exposure time to the immune phage library [26,27]. BAFF antigen was gradually reduced from 50 to 25 µg/mL to allow preferential selection of higher affinity mutants. Input/output ratio and affinity assays verified that the bio-panning and the enrichment of phages binding to BAFF were effective. To prepare crude phage to examine by monoclonal phage ELISA, 188 clones from either second or third round were chosen at random. More than half of the clones selected from the second and third round displayed positive results. Of these, 21 positive clones were collected randomly for DNA sequencing, which showed that the clones fell into different families according to sequence. Three different clones (clones 64, 52 and G3) were selected for expression of soluble periplasmic VHH and were transformed into *Escherichia coli* TG1. The products of sdAbs (anti-BAFF64, anti-BAFF52 and anti-BAFFG3) were shown to be highly pure and homogeneous by SDS-PAGE (more than 95% pure, as estimated by absorbance scanning), where the purified sdAbs appeared as a single band of 15 kDa.

The three selected sdAbs, anti-BAFF64, anti-BAFF52 and anti-BAFFG3, possess unique characteristics in terms of their different CDR structures. The three sdAbs show more than 80% sequence identity with human VH domain; the differences in amino-acid sequence between the three alpaca VHH and human VH are localized in the CDR regions. As we all know, CDR provides complementarity of paratope and epitope surfaces, and this determines the antigen-recognizing capacity of apical regions of the variable domains, defining the antibody–antigen interaction. SdAbs consist of three distinct hyper-variable regions and CDRs. A long CDR3 results in new antigen binding modes, such as binding the active site of enzymes, and also covers the hydrophobic interface that would be formed with the variable light chain domain [28,29]. These appear to enhance both the stability and relative affinity of sdAbs. The three sdAbs recognize close but distinct epitopes on BAFF and bind with high affinity. The relative affinity constant for the three sdAbs against BAFF were calculated using Bio-Layer Interferometry. The range of mean Kaff values for the three sdAbs was 10^−6^ to 10^−7^ M; anti-BAFF64 has a higher affinity than the other two sdAbs.

Importantly, the functionality of VHH was demonstrated. They can recognize BAFF molecules and were able to disturb binding between BAFF and its receptors. Anti-BAFF64 inhibited the binding of TACI to BAFF, while anti-BAFFG3 could block BMCA binding. The ratios of inhibition were 31% and 33%, respectively. TACI and BCMA are expressed by B cells at various times during their ontogeny. It has been reported that most autoantibody-producing plasma cells derive from extrafollicular foci and that their survival is largely dependent on TACI and BCMA [30]. Anti-BAFF64 could significantly prevent the combination of BAFF and TACI, which makes it a good candidate for use in bi-specific formats for therapeutic use.

Finally, differences in the degree of inhibition by the three sdAbs may be due to the differences in their affinity for BAFF. Our functional assay showed that the three sdAbs could, in a dose dependent manner, inhibit BAFF-induced B-cell lymphoma cell proliferation and anti-BAFF64 sdAb, which had a higher affinity and was the most potent in inhibiting tumor cell proliferation. Anti-BAFF64 presents a potential therapeutic approach for growth inhibition of tumor cells and must bind at a site critical to BAFF function, as it had the greatest inhibitory effect *in vitro*. In fact, because the exact nature of the three sdAbs is not fully understood, determination of their *in vivo* activity is of interest.

## 4. Experimental Section

### 4.1. Alpaca Immunization

A young female alpaca was intramuscularly inoculated with BAFF (0.2 mg/0.2 mL), and booster treatments were administered 3, 6 and 9 weeks later. For the first injection, the antigen was mixed with an equal volume of complete Freund’s adjuvant (Sigma-Aldrich, St. Louis, MO, USA), and all subsequent boosts were with incomplete Freund’s adjuvant (Sigma-Aldrich). Pre-immune sera were obtained from the jugular vein before boosts and immune sera were taken after boosts one to two weeks to test the titer. One week after the last injection, 50 mL of immune sera was collected for total RNA purification.

### 4.2. Measurement of Antibody Titers

The titer of anti-BAFF antibody was measured by ELISA. First, 96-well plates were coated with BAFF (10 µg/mL) and incubated overnight at 4 °C. Wells were then blocked with 2% skimmed milk powder in phosphate-buffered saline (MPBS), and incubated at 37 °C for 2 h. Sera were diluted with 2% MPBS and added 100 μL/well, and incubated at 37 °C for 1 h. Then the wells were added horseradish peroxidase (HRP)-labeled goat anti-alpaca antibodies after three washes with PBS containing 0.05% Tween-20 (PBST, pH 7.4), and incubated at 37 °C for 1 h. The wells were added substrate 3,3',5,5'-Tetramethylbenzidine (TMB) after three washes with PBST, and incubated at room temperature for 10 min. The absorbance of each sample was read at 450 nm with an ELISA reader.

### 4.3. Antigen Biotinylation

Antigen was biotinylated with EZ-Link Sulfo–NHS–Biotin (sulfosuccinimidobiotin, Thermo Fischer Scientific, Waltham, MA, USA) dissolved in dimethylsulfoxide (1.0 g/L). The antigen was reacted with a 10:1 molar excess of NHS–LC–Biotin buffer (Succinimidyl 6-(biotinamido)-hexanoate, Thermo Fischer Scientific, Waltham, MA, USA). After 30 min at room temperature, the biotinylated antigen was dialyzed against PBS overnight.

### 4.4. Construction of Immunized Alpaca sdAb Library

White blood cells were isolated from alpaca blood with anticoagulant using the recommended protocol and total RNA was extracted from leukocytes using the QIAamp RNA Blood Mini™ Kit (QIAGEN, Hilden, Germany). The concentration of RNA was calculated from A260 measurements. cDNA was synthesized from 1.0 µg of total RNA using a First-Strand cDNA Synthesis Kit (Invitrogen, Carlsbad, CA, USA). The amplification of VHH was performed using nested PCR. In the first PCR, the amplified region ranged from the VHH domain to the CH2 domain. Sequences of the first-PCR primers are as follows: YTCh-1 5'-CGCCATCAAGGTACCAGTTGA-3'; YT1 BN 5'-GCCCAGCCGGCCATGGCCSMKGTRCAGCTGGTGGAKTCTGGGGGAG-3'. Amplified heavy domain antibodies (around 500 bp) were purified from agarose gels using the QIAquick Gel Extraction™ kit (QIAGEN), which was then subjected to the second round of PCR. The specific degenerate *VHH* primers were used to amplify *VHH* genes (5'-CATGTGTAGATTCCTGGCCGGCCTGGCCTGAGGAGACGGTGACCT GG-3' and 3'-CATGTGCATGGCCTAGACTCGCGGCCCAGCCGGCCATGGCC-5'). The amplified product incorporated a SfiI restriction site at the ends of *VHH* genes. *VHH* genes were purified with the QIAquick PCR Purification kit (QIAGEN) and were digested and ligated into phage vector pHEN-6 [31], using a ratio of 800 ng of vector to 70 ng of VHH (which should provide the optimal vector-to-VHH ratio). The recombinant plasmids were introduced into *Escherichia coli* strain TG1 by electroporation. A small amount of electroporated cells was diluted to determine the recombination rate and the remaining cells were incubated with 2YT/Amp at 37 °C. M13KO7 helper phage was added at a 20:1 ratio, allow for infection by incubation at 37 °C without shaking for 30 min. Phage was precipitated with PEG/NaCl and the phage titer was calculated.

### 4.5. Panning the Immunized Alpaca sdAb Library

Affinity selection for BAFF-binding recombinant phages was performed [32]. First, 100 μL of M-280 streptavidin beads (Invitrogen) and 1 mL of 2% MPBS were added to a 1.5 mL microcentrifuge tube. This tube was then incubated with end-over-end rotation for 1 h at room temperature to block non-specific binding. The beads were drawn to one side of the tube with a magnetic tube holder (Dynal Biotech, Oslo, Norway) and the supernatant was discarded. Biotinylated BAFF antigen (500 nM) was mixed with the magnetic streptavidin beads and incubated with end-over-end rotation for a further 30 min at room temperature. Then, 5 × 10^11^ t.u. phage were incubated with antigen-coated magnetic streptavidin beads for 2 h at room temperature. Meanwhile, 1–2 clones from a fresh *Escherichia coli* plate were inoculated into 10 mL of 2YT, which was incubated at 37 °C and shaken at 250 rpm for 2~4 h until OD600 = 0.5. Unbound phage was discarded and the magnetic streptavidin beads were rinsed 5–10 times with 350 μL of PBST and then 5–10 times with 350 μL of PBS. Bound phage was eluted with 200 μL of freshly diluted triethylamine (TEA) (14 μL triethylamine + 1 mL distilled water), and then, the TEA was neutralized with 400 μL of 1 M Tris–HCl. TG1 culture (2 mL, OD600 = 0.5) was infected with 600 μL of eluted phage while standing at 37 °C for 30 min. Serial dilutions of infected cells were used to titer phages and the remainder was used to amplify the phage for further selection or analysis. Phages were purified with PEG (20% PEG6000–2.5 M NaCl). Three rounds of selection were carried out and populations from the second and third rounds were tested for specificity to BAFF by phage ELISA.

### 4.6. Phage Enzyme-Linked Immunosorbent Assay (ELISA)

After the second and third rounds of screening of anti-BAFF sdAb clones by phage ELISA, 188 clones were chosen at random. Bacteria from the second and third rounds of selection were diluted and plated on an LB/ampicillin plate to produce an isolated colony. The single colony was chose and cultured in 96-well plates at 37 °C for 4 to 5 h with shaking at 100 rpm. M13K07 helper phage was added and incubated at 37 °C for 30 min without shaking. Next, 5 μL ampicillin (100 mg/mL) and 15 μL kanamycin (50 mg/mL) was mixed with 50 mL 2× yeast extract-tryptone broth (2YT; composition per liter of distilled water: Tryptone 16;Yeast extract; NaCl 5 g) and added to each well before incubating overnight at 37 °C. Supernatant was collected and added to an ELISA plate, and the standard ELISA process was followed using an HRP-anti-M13 anti-body (GE Healthcare, Munich, Germany). The plate was analyzed at 450 nm with an ELISA reader. Positive phage clones were analyzed by DNA sequencing.

### 4.7. Expression and Purification of Anti-BAFF VHHs

VHHs were digested and ligated into the phage expression vector pSJF2. The recombinant plasmids were introduced into TG1 by electroporation. Individual clones were inoculated into 10 mL 2YT (supplemented with ampicillin (100 mg/mL) and grown overnight at 37 °C while shaking at 250 rpm. Soluble proteins were purified by nickel affinity using Ni-NTA (QIAGEN) as recommended by the manufacturer).

The sdAbs were assessed by size exclusion chromatography using a Superdex^75^ (GE Healthcare Bio-Sciences AB) column on an AKTA purifier 2000 system according to the manufacturer’s instructions. Size exclusion chromatography was carried out in 0.1 mol/L phosphate buffer at a flow rate of 0.8 mL/min. Molecular weight was determined using the Gel Filtration Calibration Kit HMW (high molecular weight, GE healthcare). Additionally, marker retention volumes were incorporated into the top of all chromatographs (Figure 2B,D,F). Purified proteins were also assessed by SDS-PAGE, and the protein concentrations were determined by BCA (Pierce, Rockford, IL, USA).

### 4.8. Affinity Measurements

The relative affinity constant of each sdAb against BAFF was calculated by BLI. The BAFF antigen was biotinylated using NHS–LC–Biotin buffer at a 3:1 molar ratio of biotin to protein for 30 min at room temperature, followed by dialysis against PBS. Binding assays were performed by BLI using an Octet RED system (Pall Forte Bio Europe, Portsmouth, UK) and Fortebio Acquisition Software in 96-well microtiter plates at room temperature with orbital sensor agitation of 1000 rpm. Biotinylated BAFF (20 μg/mL) was load onto Streptavidin (SA) biosensors, a baseline was established in PBST buffer (PBS pH 7.4, 0.1% BSA, albumin fraction and 0.02% Tween-20, both Merck KGaA, Darmstadt, Germany) prior to association at varying analyte concentrations. Serial dilutions were made to produce 200 μL of 20, 10, 5, 2.5 and 1.25 µM sdAbs in appropriate wells of the assay plate. Antigen-coated sensors were placed in five different concentrations of sdAbs for 300 s, during which antigen was bound by antibodies on the sensor. Sensors were rinsed in kinetics buffer for 1500 s, which also served as the background buffer. Octet Analysis Software version 6.4 was used for automatic data processing. Biosensor data were fit using a 1:1 binding model.

### 4.9. ELISA Competition Assays

First, two 96-well plates were coated with 100 µL BAFF (10 µg/mL in 50 mM NaCO_3_ buffer, pH 9.5) and incubated overnight at 4 °C, and unspecific binding was blocked by incubation with 380 µL of 3% BSA per well at 37 °C for 2 h. Then each of the three individual sdAbs (anti-BAFF64, anti-BAFF52 and anti-BAFFG3) fused to 50 µL BCMA (50 µg/mL), at various dilutions (0, 25, 50, 100, 200, 500 µg/mL) with 3% BSA, were added in the wells of one plate for 2 h at 37 °C. And in the other plate, each of the sdAbs against BAFF (64, 52 and G3) with different concentration (0, 5, 10, 20, 50, 100 µg/mL) was incubate with 50 µL TACI (10 µg/mL) for 2 h at 37 °C, and the total volume of each solution was 100 µL. Anti-CD20 sdAb was the negative control. Wells were washed three times with PBST, after which 100 µL of the HRP-labeled goat anti-human IgG (Sigma-Aldrich) were added and incubated for 1 h at room temperature. The wells were added substrate TMB after three washes with PBST, and incubated at room temperature for 10 min. Optical density was read at 450 nm.

### 4.10. Raji Cells Proliferation Inhibition Assay

Raji Cells (American Type Culture Collection, Manassas, VA, USA) were adjusted to 10^6^ cells/mL and cultured in 96-well flat-bottom plates with 100 µL RPMI-1640 (GibcoBRL, Gaithersburg, MD, USA) per well supplemented with 10% FBS (Sigma-Aldrich). BAFF (750 ng/mL final concentration) was added to each well, and the cells were incubated at 37 °C in a humidified atmosphere with 5% CO_2_. After 48 h, the three sdAbs were added to wells to achieve different concentrations (0, 25, 50, 75 and 100 µg/mL). Raji Cells were incubated for a total of 72 h and all assays were carried out in triplicate. Cell proliferation was measured with a Cell Counting Kit-8 (Dojindo, Kumamoto, Japan).

## 5. Conclusions

Our previous work demonstrated that the success of this new generation of anti-BAFF sdAbs will depend strongly on multiple factors, including library diversity and size, the affinity and immunogenicity of the resultant antibody, and of course the effect of the targeted molecule on cancer cells. These three anti-BAFF sdAbs may offer new choices when targeting tumors for B cell lymphoma diagnosis or therapy.
